# Differentiation between glioblastoma and solitary brain metastases using perfusion and amide proton transfer weighted MRI

**DOI:** 10.3389/fnins.2025.1533799

**Published:** 2025-02-05

**Authors:** Malte Knutsson, Tim Salomonsson, Faris Durmo, Emelie Ryd Johansson, Anina Seidemo, Jimmy Lätt, Anna Rydelius, Sara Kinhult, Elisabet Englund, Johan Bengzon, Peter C. M. van Zijl, Linda Knutsson, Pia C. Sundgren

**Affiliations:** ^1^Department of Clinical Sciences, Division of Radiology, Lund University, Lund, Sweden; ^2^Department of Medical Imaging and Physiology, Skåne University Hospital, Lund, Sweden; ^3^Department of Clinical Sciences, Division of Neurology, Lund University, Lund, Sweden; ^4^Department of Clinical Sciences, Division of Oncology, Lund University, Lund, Sweden; ^5^Department of Clinical Sciences, Division of Pathology, Lund University, Lund, Sweden; ^6^Department of Clinical Sciences, Division of Neurosurgery, Lund University, Lund, Sweden; ^7^F.M. Kirby Research Center for Functional Brain Imaging, Kennedy Krieger Institute, Baltimore, MD, United States; ^8^Department of Radiology, Johns Hopkins University School of Medicine, Baltimore, MD, United States; ^9^Department of Neurology, Johns Hopkins University School of Medicine, Baltimore, MD, United States; ^10^Department of Medical Radiation Physics, Lund University, Lund, Sweden; ^11^LBIC, Lund University Bioimaging Center, Lund University, Lund, Sweden

**Keywords:** glioma, metastases, CEST, amide proton transfer-weighted imaging, dynamic susceptibility contrast MRI

## Abstract

**Objectives:**

Early diagnostic separation between glioblastoma (GBM) and solitary metastases (MET) is important for patient management but remains challenging when based on imaging only. The objective of this study was to assess whether amide proton transfer weighted (APTw) MRI alone or combined with dynamic susceptibility contrast (DSC) MRI parameters, including cerebral blood volume (CBV), cerebral blood flow (CBF), and leakage parameter (K2) measurements, can differentiate GBM from MET.

**Methods:**

APTw MRI and DSC-MRI were performed on 18 patients diagnosed with GBM (*N* = 10) or MET (*N* = 8). Quantitative parameter maps were calculated, and regions-of-interest (ROIs) were placed in whole tumor, contrast-enhanced tumor (ET), edema, necrosis and normal-appearing white matter (NAWM). The mean and max of the APTw signal, CBF, leakage-corrected CBV and K2 were obtained from each ROI. Except for K2, all were normalized to NAWM (nAPTw_mean/max_, nCBF_mean/max_, ncCBV_mean/max,_). Receiver Operating Characteristic (ROC) curves and area-under-the-curve (AUC) were assessed for different parameter combinations. Statistical analyses were performed using Mann–Whitney U test.

**Results:**

When comparing GBM to MET, nAPT_max_, nCBF_max_, ncCBV_max_ and ncCBV_mean_ were significantly increased (*p* < 0.05) in ET with AUC being 0.81, 0.83, 0.85, and 0.83, respectively. Combinations of nAPTw_max_ + ncCBV_max_, nAPTw_mean_ + ncCBV_mean_, nAPTw_max_ + nCBF_max_, nAPTw_max_ + K2_max_ and nAPTw_max_ + ncCBV_max_ + K2_max_ in ET showed significant prediction in differentiating GBM and MET (AUC = 0.92, 0.82, 0.92, 0.85, and 0.92 respectively).

**Conclusion:**

When assessed in Gd-enhanced tumor areas, nAPTw MRI signal intensity alone or combined with DSC-MRI parameters, was an excellent predictor for differentiating GBM and MET. However, the small cohort warrants future studies.

## 1 Introduction

Being diagnosed with a brain tumor is often a life-threatening event, given that this disease is one of the most lethal forms of cancer, which poses a significant medical challenge (Siegel et al., 2023). The majority of brain tumors are metastases (American Association of Neurological Surgeons, 2023), while the most prevalent type of primary brain tumor is glioma with glioblastoma (GBM) being the most common and aggressive, classified grade 4 according to the World Health Organization (WHO) (Louis et al., 2021). Given their different management strategies, accurate differentiation between GBM and solitary metastases (MET) is crucial for the clinical outcome. Particularly, early diagnosis of suspected metastases without an established primary cancer site may significantly affect patient management, depending on whether the histopathological diagnosis turns out to be a primary brain tumor or metastases (Campos et al., 2009).

MRI is the standard modality for brain tumor diagnosis, treatment follow-up, stereotactic biopsies, surgical resection strategies, and differentiation between post-treatment effects and recurrent tumor growth. Although multiple MRI methods have been introduced over the years, diagnostic separation between GBM and solitary metastases has remained challenging. For instance, both GBM and MET may be hyperintense on gadolinium (Gd)-enhanced T1-weighted MRI, with hyperintense tumor and edema on T2-weighted MR images. Thus, exploration of more physiological or molecular oriented imaging approaches is essential. Since angiogenesis forms new tumor vessels, one such technique is perfusion weighted MRI for assessing malignancy and monitoring the effects of treatment (Law et al., 2004; Boxerman et al., 2006; Law et al., 2006; Geer et al., 2012). A common perfusion method is dynamic susceptibility contrast (DSC) MRI, where a rapid intravenous injection of a Gd contrast agent is performed during dynamic T2/T2*-weighted imaging (Rempp et al., 1994; Boxerman et al., 2006; Knutsson et al., 2010). It has been shown that DSC-MRI is a valuable tool for distinguishing GBM from MET, since GBM, as a result of an elevated cell proliferation, has an increased cerebral blood volume (CBV) in comparison to MET, especially in the peritumoral zone (Server et al., 2011; She et al., 2019). However, when the blood brain barrier (BBB) is disrupted, quantification of CBV will be erroneous due to Gd leakage into the tumor extravascular extracellular space (EES). Correcting for leakage (cCBV), which in the process also determines a leakage parameter K2, has shown to better asses the glioma grade (Boxerman et al., 2006). Compared to MET, GBM has a more infiltrative nature with a disrupted BBB, making the leakage parameter K2 a suitable additional measure in distinguishing these tumor types (Server et al., 2011).

Another promising method for brain tumor imaging is amide proton transfer weighted (APTw) MRI. This chemical exchange saturation transfer (CEST) MRI technique has a contrast that in large part originates from the amide protons in mobile cellular proteins and peptides and can be measured indirectly through the water signal (Zhou et al., 2003a; Zhou et al., 2003b; Zhou et al., 2019). APTw MRI of brain tumors has allowed the differentiation between high and low-grade gliomas (Bai et al., 2017; Jiang et al., 2017a; Zou et al., 2018; Su et al., 2021; Zhang et al., 2021) and separating recurrent tumor from treatment necrosis (Ma et al., 2016; Jiang et al., 2019). A few studies have investigated if APTw MRI also has the capability to separate GBM from MET. Yu et al. (2017) showed a high accuracy when measuring the mean APTw (APTw_mean_) signal in the peritumoral region, but no significant difference was found within the tumor region itself. Another APTw MRI study, however, showed no significance in distinguishing these tumor types for peritumoral regions (Kamimura et al., 2019). To improve diagnostic performance for distinguishing GBM from MET, other MRI modalities have been combined with APTw MRI. For example, a study by Chen et al. (2023) combining cerebral blood flow (CBF), obtained from arterial spin labeling (ASL), and APTw increased accuracy when assessing the peritumoral region. To our knowledge, no prior study has assessed whether APTw MRI, in combination with CBV, CBF, and K2 obtained from DSC-MRI, can improve differentiation between GBM and MET. As such, the objective of this study was to investigate the diagnostic performance of APTw MRI and DSC-MRI, individually and in combination, in distinguishing GBM from MET.

## 2 Materials and methods

### 2.1 Patients

Eighteen patients (average age 59 years, 8 females) diagnosed with GBM (*N* = 10) or MET (*N* = 8) who met the inclusion criteria between July 2017 and May 2021 were included in this retrospective study. The inclusion criteria were age ≥ 18 and previous routine computed tomography (CT) or MRI revealing a suspected brain neoplasm. Additional inclusion criteria in the present study were surgery or biopsy, with retrospective histopathological diagnosis as GBM or MET according to WHO 2021 classification. Seven of the patients had previously been included in a separate study investigating the radiological value of APTw MRI in differentiating low-grade glioma from high-grade glioma (Durmo et al., 2020). The current study, however, is based on a very different scientific hypothesis. Furthermore, APTw data were post-processed using different software tools. The project was approved by the Swedish Ethical Review Authority, and written informed consent was obtained from each participant.

### 2.2 MRI acquisition protocol

Patients were examined on a 3 T MAGNETOM Prisma scanner with a 20-channel head coil (Siemens Healthcare, Erlangen, Germany) with a total scan time for the protocol being approximately 45 min. Pre-and post-contrast-enhanced T1 magnetization prepared rapid gradient echo (MPRAGE), fluid attenuated inversion recovery (FLAIR), T2 Turbo spin-echo (TSE), APTw and DSC images were acquired during the same session. Sequence parameters can be found in Table 1.

**Table 1:** Parameters of the MRI sequences.

	TR (ms)	TE (ms)	FA (°)	FOV (mm^2^)	Acq. matrix	Slice thickness (mm)	TA (min)
T1-MPRAGE*	1,900	2.54	9	256×256	256×256	1	5:13
FLAIR**	5,000	393	90	256×256	256×256	1	4:25
T2 TSE	6,000	100	90	256×256	256×256	5	2:02
APTw-MRI	10	2.71	12	208×256	104×128	4	6:50
DSC-MRI	1,243	29	60	220×220	128×128	5	1:30

TR, repetition time; TE, echo time; FOV, field of view; Acq. Acquisition; TA, acquisition time. *Inversion time of 900 ms. **Inversion time of 1,800 ms.

A prototype CEST 3D GRE MRI sequence from the vendor was used to acquire 22 slices of APTw images. The saturation module consisted of 5 hyperbolic secant pulses of 100 ms with 4 interpulse delays (61 ms) using a B_1_ of 2 μT. Water saturation spectral (Z-spectral) acquisition was obtained by applying the saturation module at 21 frequency offsets over a range of ±5 ppm relative to the whole-brain water resonance frequency in 0.5 ppm steps. An unsaturated reference image (S_0_) was acquired at -150 ppm to minimize magnetization transfer effects from semisolid macromolecules. To avoid T1-relaxation effects from Gd on the saturation, APTw imaging was performed before injection of the agent.

DSC-MRI was performed using a single-shot gradient echo-echo planar imaging (GRE-EPI) sequence, acquiring 20 slices. The Gd-based contrast agents Clariscan® (Marlborough, MA, GE HealthCare) and Dotarem® (Billdal, Sweden, Gothia Medical) were administered to 12 and 6 patients, respectively, with a target dose of 0.1 mmol/kg and injection rate of 5 mL/s, approximately 15 s after the start of the dynamic series. The contrast agent bolus was followed by a 20-mL saline flush, injected at a rate of 5 mL/s.

### 2.3 Postprocessing

#### 2.3.1 Amide proton transfer weighted imaging

A prototype software developed by Olea Medical® (Olea Medical Solutions, La Ciotat, France) was used to process the data and retrieve APTw maps. Z-spectral intensities were calculated by normalizing the water signal intensities (S_sat_) at each saturation frequency offset ( Δω ) to the intensity without saturation (S_0_). Voxel-based B_0_ correction was performed by shifting the minimum of the Z-spectrum to 0 ppm. APTw (%) was calculated using:


(1) $$\mathrm{APTw}\left(\%\right)=\frac{\int_{-\Delta \omega_{2}}^{-\Delta \omega_{1}}\tilde{Z}\left(\Delta \omega \right)d\omega -\int_{\Delta \omega_{1}}^{\Delta \omega_{2}}\tilde{Z}\left(\Delta \omega \right)d\omega }{\Delta \omega_{2}-\Delta \omega_{1}}\text{,}$$

where $\tilde{Z}$ is the linear-interpolated Z-spectrum. $\Delta \omega_{1}$ and $\Delta \omega_{2}$ are the offset boundaries (3.1 ppm and 3.9 ppm, respectively). Reduction of fluid artifacts was also performed by applying fluid suppression (FS), creating an additional parameter, APTw_FS_ given by Schure et al. (2024):


(2) $${\mathrm{APTw}}_{FS}=\mathrm{APTw}\frac{{\sigma^{’}}_{WM}^{2}\;}{Z_{ref}^{2}}\text{,}$$

where Z_ref_ is the signal intensity at offset frequency -3.5 ppm and the σ’_WM_ correction factor is set to the Z-spectrum intensity at -3.5 ppm in white matter (WM). To obtain σ’_WM_, we performed Bloch-McConnell simulations using the sequence parameters from the APTw protocol and WM_3T_001_bmsim.yaml from the pulseq-sim library, https://github.com/pulseq-cest/pulseq-cest-library/. From this, we derived σ’_WM_ to be 0.4, a value similar to 0.35 used in Schure et al. (2024), which used different APT acquisition parameters.

#### 2.3.2 Dynamic susceptibility contrast MRI

The DSC-MRI images were processed using OLEA Sphere (Olea Medical Solutions, La Ciotat, France) using standard tracer kinetic theory and a linear fitting algorithm to obtain K2 and leakage-corrected CBV (cCBV) (Boxerman et al., 2006). The arterial input function (AIF) was defined by taking the average of semi-automatically selected concentration-time curves from voxels within the middle cerebral artery (MCA) around the Sylvian fissure (Knutsson et al., 2014). To obtain CBF, deconvolution of the concentration-time curve in each voxel with the AIF was performed using delay-insensitive singular value decomposition.

#### 2.3.3 Segmentation and regions of interest (ROI)

**Figure 1:**
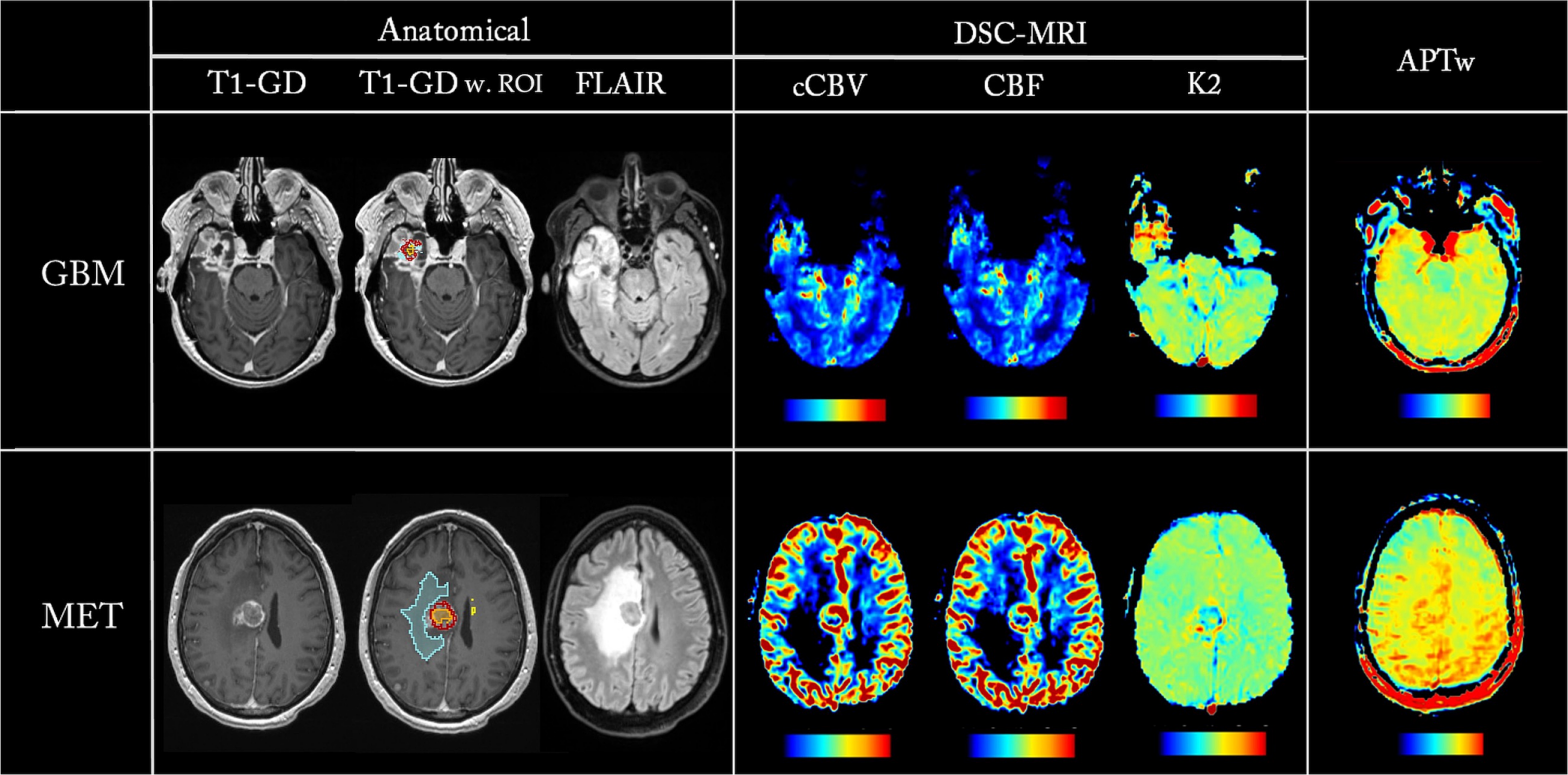
Parametric maps from one GBM patient (first row) and one MET patient (second row). ROIs are overlayed on T1-Gd, showing ET in red, necrosis in orange, and edema in blue.

The images were analyzed using 3D Slicer Segmentation Tool (Version 5.6.2^1^) (Fedorov et al., 2012). Regions of interests (ROIs) were drawn manually on the morphological T1-MPRAGE post Gd-contrast images (T1-Gd), with assistance from FLAIR and T1-MPRAGE images (T1). The ROIs were placed in: 1. The contrast-enhanced (ET) part of the lesion, described as hyperintense in the T1-Gd compared to T1, 2. The lesion’s necrotic area, described as the hypointense area within the ET, and 3. the edema, described as the hyperintense area on FLAIR excluding the ET and necrosis (Figure 1). WT was defined as the full extent of the tumor, including the ET, necrosis and edema. ROI was also placed in normal appearing white matter (NAWM) in the centrum semiovale region. Mean and max values for all parameters were obtained from the ROIs. All values, except K2, were normalized to NAWM, resulting in nAPTw_mean_, nAPTw_FS,mean_, nCBF_mean_, ncCBV_mean_ and nAPTw_max_, nAPTw_FS,max_, nCBF_max_, ncCBV_max_, respectively. Since leakage should be non-existent in healthy white matter, normalization with NAWM would result in large erroneous values in K2.

### 2.4 Statistics

Statistical analyses were performed using IBM SPSS Statistics® (IBM Corp, Armonk, NY, USA). A Mann–Whitney U test was applied when comparing the different parameters from the GBM and MET cohorts, with a *p*-value of <0.05 considered significant. The diagnostic efficacy of the parameters for distinguishing GBM from MET was evaluated by assessing Receiver Operating Characteristic (ROC) curves and area-under-the-curve (AUC). Binary logistic regression was used to evaluate the ability of different parameter combinations to distinguish GBM from MET. Since the aim was to investigate each parameter/combination independently and not risk that truly important differences may be deemed non-significant, we did not apply any multiple comparison correction (Perneger, 1998).

## 3 Results

Table 2 summarizes clinical patient characteristics. There was no statistical significance between the groups with respect to age and gender.

**Table 2:** Patient demographics.

	GBM (*n* = 10)	MET (*n* = 8)	*p*-value
Age (years)	54 ± 18	63 ± 6	0.18
Gender (male:female)	7:3	3:5	0.17
Edema	10	6	0.93
Necrosis	5	2	0.55
MGMT-unmethylated	4		
IDH-wildtype	10		

IDH, Isocitrate dehydrogenase; MGMT, O^6^-methylguanine-DNA methyl-transferase.

Figure 1 shows parametric maps from two patients, one GBM and one MET, with the corresponding ROIs overlayed on the T1-Gd. Notice that all parametric maps reveal hyperintensity in the lesion areas in conventional imaging.

Parameter values, as well as a summary of the Mann–Whitney U statistical analysis between GBM and MET for the different parameters, are shown in Table 3. For the ET region, nAPTw_max_, nCBF_max_, ncCBV_max_ and ncCBV_mean_ were significantly higher (*p* < 0.05) in GBM compared to MET. In addition, ET based nAPTw_FS,max_ and WT based ncCBV_mean_ showed a trend toward differentiating between GBM and MET (*p* = 0.06).

**Table 3:** Comparison between GBM and MET for nAPTw, nAPTw_FS_, nCBF, ncCBV, and K2 for WT, ET, Edema and Necrosis.

		GBM (*n* = 10)			MET (*n* = 8)			Mann U		
		Mean	Max	SD	Mean	Max	SD	Factor	U	*p*-value*
nAPTw	WT	1.9	12	0.67	1.4	12	0.75	Max	33	0.57
								Mean	23	0.15
	ET	2.0	4.9	0.60	1.7	4.4	0.74	**Max**	**15**	**0.03**
								Mean	24	0.17
	Edema	0.89	2.7	0.71	0.87	3.2	0.76	Max	41	1.0
								Mean	40	1.0
	Necrosis	0.76	4.4	0.86	0.24	3.2	0.45	Max	28	0.32
								Mean	27	0.27
nAPTw_FS_	WT	0.92	14	0.42	0.81	6.0	0.30	Max	40	1.0
								Mean	42	0.90
	ET	1.0	3.2	0.42	0.91	2.3	0.38	Max	18	0.06
								Mean	36	0.76
	Edema	0.44	1.6	0.38	0.43	1.7	0.36	Max	39	0.97
								Mean	38	0.90
	Necrosis	0.25	2.1	0.27	0.15	2.1	0.29	Max	29	0.36
								Mean	31	0.46
nCBF	WT	2.7	178	1.7	1.7	13	1.3	Max	21	0.17
								Mean	20	0.14
	ET	3.7	18	1.7	2.3	13	1.0	**Max**	**12**	**0.02**
								Mean	20	0.14
	Edema	1.2	7.1	0.98	0.69	8.5	0.77	Max	24	0.28
								Mean	21	0.17
	Necrosis	0.25	4.2	0.28	0.25	5.8	0.55	Max	26	0.37
								Mean	28	0.48
ncCBV	WT	3.0	23	1.7	1.6	12	1.2	Max	19	0.11
								Mean	16	0.06
	ET	4.3	23	1.8	2.4	12	0.86	**Max**	**11**	**0.02**
								**Mean**	**12**	**0.02**
	Edema	1.3	8.5	1.1	0.67	8.5	0.75	Max	23	0.24
								Mean	19	0.11
	Necrosis	0.21	2.4	0.25	0.25	5,5	0.54	Max	26	0.37
								Mean	29	0.54
K2	WT	−98	4,191	169	−243	1909	346	Max	27	0.42
								Mean	32	0.74
	ET	77	3,005	335	103	1,420	231	Max	27	0.42
								Mean	37	1.0
	Edema	5.8	1,021	238	−23	678	28	Max	23	0.84
								Mean	20	0.95
	Necrosis	24	789	140	−115	368	178	Max	1	0.19
								Mean	4	0.86

Significant differences are marked in bold. For the perfusion parameters, *n* = 9 since one patient did not undergo perfusion weighted MRI. *The statistical significance, *p*, was set to less or equal than 0.05.

**Figure 2:**
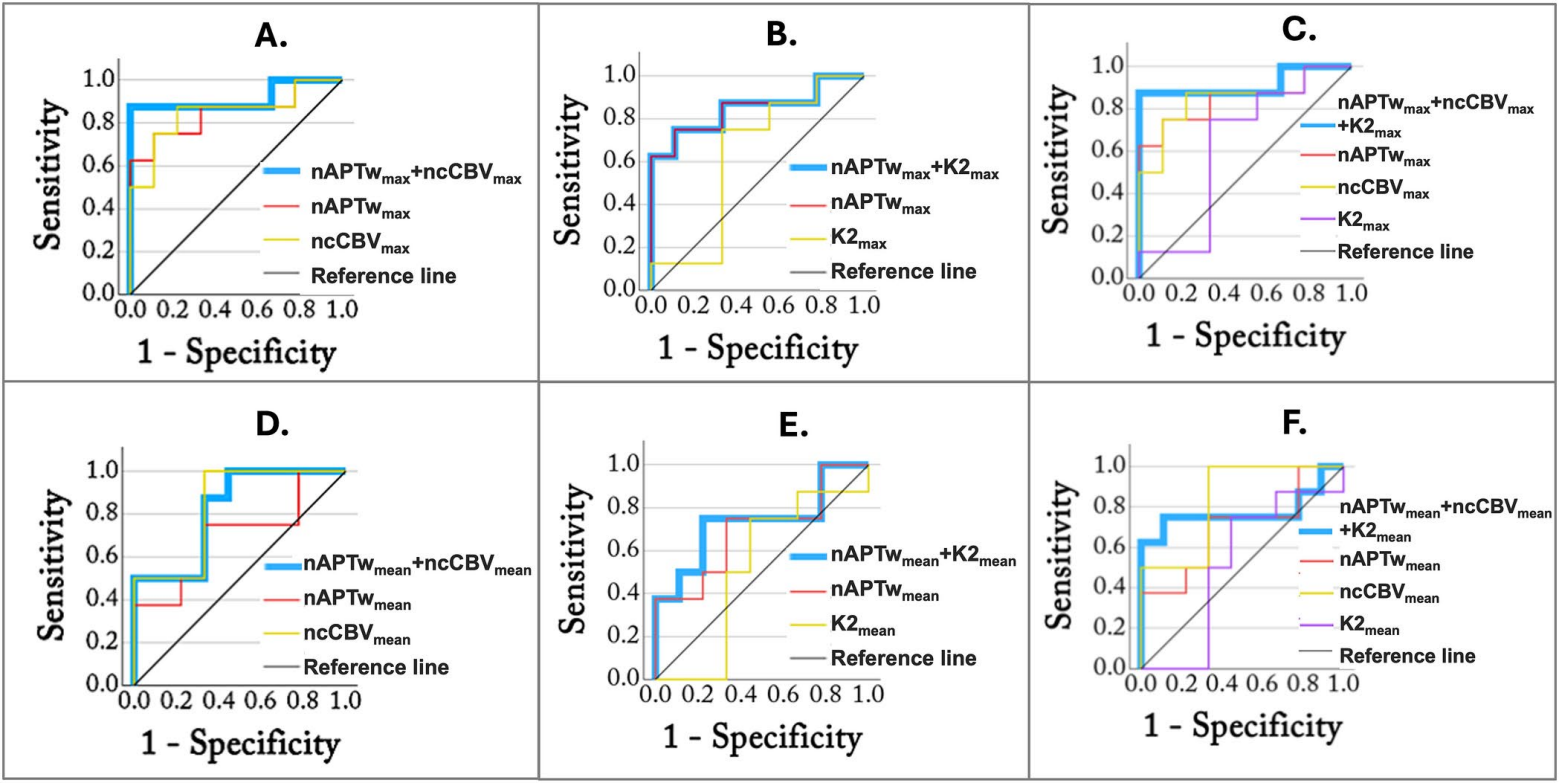
ROC analysis using combined parameters measured in the ET region. The blue lines represent combined parameters: **(A)** nAPTw_max_ + ncCBV_max_, **(B)** nAPTw_max_ + K2_max_, **(C)** nAPTw_max_ + ncCBV_max_ + K2_max_, **(D)** nAPTw_mean_ + ncCBV_mean_, **(E)** nAPTw_mean_ + K2_mean_, and **(F)** nAPTw_mean_ + ncCBV_mean_ + K2_mean_.

The results from the ROC analysis of individual parameters are summarized in Table 4, showing that nAPTw_max_, nCBF_max_, ncCBV_max_, and ncCBV_mean_ obtained from ET are the most accurate in distinguishing GBM from MET (AUC = 0.81, 0.83, 0.85, and 0.83, respectively). A combination of the parameters measured in ET, resulted in an increase in AUC for nAPTw_max_ with ncCBV_max_, nAPTw_max_ with nCBF_max_, and nAPTw_max_ with K2_max_ (AUC = 0.92, 0.92, and 0.85, respectively) (Table 5; Figure 2). Adding K2_max_ to nAPTw_max_ + ncCBV_max_ did not increase the AUC.

**Table 4:** ROC analysis of individual parameters.

Value	Segment	Parameter	AUC	Std. Error	*p-*value*	95% Confidence interval (Lower bound – Higher bound)
MAX	WT	nAPTw	0.59	0.15	0.55	0.30–0.88
		nCBF	0.71	0.13	0.12	0.45–0.97
		ncCBV	0.74	0.13	0.060	0.49–0.98
		K2	0.52	0.18	0.90	0.17–0.88
	ET	nAPTw	0.81	0.11	0.004	0.60–1.0
		nCBF	0.83	0.11	0.003	0.61–1.0
		ncCBV	0.85	0.10	0.001	0.65–1.0
		K2	0.60	0.17	0.58	0.26–0.93
	Edema	nAPTw	0.49	0.15	0.93	0.20–0.78
		nCBF	0.67	0.14	0.25	0.39–0.95
		ncCBV	0.68	0.14	0.20	0.41–0.95
		K2	0.45	0.17	0.78	0.13–0.78
MEAN	WT	nAPTw	0.71	0.13	0.11	0.45–0.97
		nCBF	0.72	0.13	0.09	0.46–0.98
		ncCBV	0.78	0.12	0.02	0.54–1.0
		K2	0.33	0.17	0.31	0.01–0.66
	ET	nAPTw	0.70	0.13	0.13	0.44–0.96
		nCBF	0.72	0.13	0.08	0.47–0.97
		ncCBV	0.83	0.10	0.001	0.63–1.0
		K2	0.33	0.17	0.33	0.00–0.67
	Edema	nAPTw	0.50	0.14	1.0	0.22–0.78
		nCBF	0.71	0.13	0.12	0.45–0.97
		ncCBV	0.74	0.13	0.07	0.48–0.99
		K2	0.52	0.18	0.90	0.17–0.88

^*^Null hypothesis true area equals 0.5. One patient from the GBM group is excluded in this analysis due to missing perfusion data.

**Table 5:** ROC analysis of combined parameters from the ET segment.

Value	Parameters	AUC	Std. Error	*p*-value*	95% Confidence interval (Lower bound – Higher bound)
MAX	nAPTw + ncCBV	0.92	0.08	0.000	0.76–1.1
	nAPTw + nCBF	0.92	0.08	0.000	0.76–1.1
	nAPTw + K2	0.85	0.10	0.001	0.65–1.0
	nAPTw + ncCBV + K2	0.92	0.08	0.000	0.76–1.1
MEAN	nAPTw + ncCBV	0.82	0.10	0.002	0.62–1.0
	nAPTw + nCBF	0.75	0.12	0.040	0.51–0.99
	nAPTw + K2	0.74	0.13	0.069	0.48–0.99
	nAPTw + ncCBV + K2	0.78	0.13	0.035	0.52–1.0

^*^Null hypothesis true area equals 0.5. One patient from the GBM group is excluded in this analysis due to missing perfusion data.

## 4 Discussion

In this study we investigated if APTw MRI alone or combined with DSC-MRI can differentiate glioblastomas from solitary brain metastases. We showed that the nAPTw_max_, nCBF_max_, ncCBV_max_ and ncCBV_mean_ in enhancing tumor regions were significantly different in GBM compared to MET.

Similar to our study, the parameter APTw_max_ has previously been shown to be more accurate than APTw_mean_ in distinguishing IDH mutational status and low-grade from high-grade glioma and predicting IDH mutation status (Jiang et al., 2017b; Durmo et al., 2020). When comparing APTw measures in different regions, we found that nAPTw_max_ in the ET region was the most valuable in distinguishing the two tumor types, while the peritumoral region showed no significant difference. This is in accordance with a previous study by Kamimura et al. (2019). However, these findings contradict Yu et al. (2017) where a significant difference in the APTw signal intensity in the peritumoral edema between GBM and MET was demonstrated. Since GBM is by nature invasive, infiltrating GBM tumor cells are expected to be present in the peritumoral edema (Patel et al., 2024), which in theory should result in a higher APTw signal compared to MET (Yu et al., 2017; Patel et al., 2024). Notably, Yu et al. used a voxel size of 1.65 × 3.15 × 6.00 mm^3^, while the voxel size in our study was 2 × 2 × 4 mm^3^ close to a 50% reduction of the volume. A larger amount of partial volume effects may lead to tissue mixing with the enhanced tumor region and thus an increase of the APTw signal in the peritumoral edema. In addition, our sequence has a short saturation time, which has previously been shown to enhance contributions from proteins in the blood vessels (Durmo et al., 2020), which may explain the significance only in Gd-enhanced areas. Other differences between these studies were that the whole lesion area was segmented in our study while the five smaller ROIs in Yu et al. did not cover the full image abnormality. We also calculated the APTw value using the integral over multiple frequency points instead of a single Z-spectral value at 3.5 ppm (Equation 1).

APTw using fluid suppression reduces contributions from protein-containing fluidic compartments in tumors (e.g., liquefactive necrosis) by utilizing a correction factor. As can be seen from Equation 2, any region with a Z_ref_ value higher than the correction factor will lead to APTw_FS_ being lower than APTw. Tissues with long T2, such as CSF, and liquefactive necrosis, will have a narrow spectral linewidth and thus a decreased APTw_FS_. In our study, nAPTw_FS_ was clearly reduced compared to nAPTw in all lesion regions. However, contrary to nAPTw, nAPTw_FS_ did not show any statistical significance in distinguishing MET and GBM in ET. One reason for this can be that our APTw maps are more weighted for blood vessels as explained above and supported by the measured large decrease in nAPTw values when applying the fluid-suppression, while the APTw signal at higher saturation strength has a large semisolid component that is not removed by fluid correction. Another reason can be that the correction factor was set to be the same in all patients. Ideally, this factor should be the Z_ref_ value in WM at -3.5 ppm in each individual patient. This can be achieved by measuring the Z-spectra value in WM for each patient since the use of fluid suppression should not alter the WM APTw intensity. However, we observed a difference between APTw_FS_ and APTw values in NAWM, and this may explain the lower statistical significance in nAPTw_FS_ in ET. Therefore, individual correction factors may be of value (Schure et al., 2024), and in future studies, we aim to incorporate this in the APTw_FS_ processing.

From DSC-MRI we found that nCBF_max_, ncCBV_max_ and ncCBV_mean_ measured in ET were significantly higher in GBM than MET for the Gd-enhanced tumor region. However, we found no statistical significance for peritumoral edema, while other studies have shown this for both nCBV and ncCBV (Server et al., 2011; She et al., 2019; Li et al., 2020). It is worth noting that in accordance with these studies, our mean/max values were higher in the peritumoral edema in GBM compared to MET (4.3, 24 vs. 2.4, 12). K2 (both mean and max) did not show any significant differences between GBM and MET and did not improve the diagnostic accuracy. There have been conflicting results between studies as K2_mean/max_ showed promise to distinguish GBM from MET in one study (Server et al., 2011), but with less accuracy than nCBF and ncCBV, while another study showed no statistical difference between the tumor types using K2_mean_ (Toh et al., 2014). One issue with K2 is that the leakage can produce both an increase in signal due to increased T1 relaxation or a decrease due to increased T2* relaxation during and after Gd bolus passage. Thus, depending on the imaging parameters used, these effects can in principle cancel each other out making K2 quantification difficult (Elschot et al., 2023).

Previously, it was shown that combining APTw signal intensity and CBF (obtained from ASL MRI), increased the diagnostic efficiency for separating GBM and MET (Chen et al., 2023). In this study we used DSC-MRI, which has the advantage over ASL of larger contrast-to-noise and providing additional parameters (cCBV and K2). The goal was to find if any parameter combination could improve the diagnostic performance, thus warranting an additional method in the MRI examination. In ET, APTw_max_ alone had an AUC = 0.81 comparable to ncCBV_max_ with AUC = 0.85. Combining these, AUC increased to 0.92. Thus, the diagnostic accuracy increases substantially by combining APTw and DSC-MRI but with the additional drawback of having to do a Gd injection.

There are some limitations to this study. First, the small cohort size limits the ability to generalize the findings. Therefore, future studies with larger sample sizes are warranted. Second, the MET group was heterogeneous, with lesions arising from lung adenocarcinoma and malignant melanoma. It has been shown that different histological types of metastases from the same primary site of origin may show different levels of APTw signal, for example squamous cell carcinoma and adenocarcinoma of the lung (Xiang et al., 2024). Therefore, a homogeneous cohort, including solitary brain metastases from the same primary site of origin and histology, may present more similar APTw values and thus enable improved separation from gliomas. Third, due to hardware limitations, the APTw sequence had a shorter total saturation time than recommended in a recent consensus paper (Zhou et al., 2022). This resulted in a lower contrast-to-noise, increased contribution from blood vessels (high in protein), and reduced contribution from asymmetry in the semisolid tissue contribution. Although careful review of the placement of the ROI was performed to reduce the risk of including obvious blood vessels in the ROI, partial volume effects might still be present.

In conclusion, differentiating GBM from MET is of significant clinical value in decision-making and patient management. This study shows that APTw MRI and DSC-MRI are valuable tools in differentiating between GBM and MET. In addition, the combination of APTw MRI and DSC-MRI further increases the ability to distinguish the two tumor types using the Gd-enhanced tumor region.

## Data Availability

The raw data supporting the conclusions of this article will be made available by the authors, without undue reservation.

## Footnotes

^1^ Slicer.org
